# A Decade Experience on Severe Combined Immunodeficiency Phenotype in Oman, Bridging to Newborn Screening

**DOI:** 10.3389/fimmu.2020.623199

**Published:** 2021-01-15

**Authors:** Nashat Al Sukaiti, Khwater Ahmed, Jalila Alshekaili, Mahmood Al Kindi, Matthew C. Cook, Tariq Al Farsi

**Affiliations:** ^1^ Department of Pediatric Allergy and Clinical Immunology, The Royal Hospital, Muscat, Oman; ^2^ Department of Microbiology and Immunology, Sultan Qaboos University Hospital, Muscat, Oman; ^3^ Department of Immunology and Infectious Disease, John Curtin School of Medical Research, Australian National University, Canberra, NSW, Australia; ^4^ Translational Research Unit, Department of Immunology, The Canberra Hospital, Canberra, NSW, Australia; ^5^ Centre for Personalized Immunology (NHMRC Centre of Research Excellence), John Curtin School of Medical Research, Australian National University, Canberra, NSW, Australia

**Keywords:** severe combined immunodeficiency, children, lymphopenia, newborn screening, hematopoietic stem cell transplantation, Omani

## Abstract

**Introduction:**

Severe combined immunodeficiency (SCID) results from various monogenic defects that impair immune function and brings on early severe and life-threatening infections. The main stay of treatment for SCID is hematopoietic stem cell transplant (HSCT) with near normal survival at 5 years for an early transplant done at or before the age of 3.5 months of life and the patient is maintained free of infections. Although overall rare, it constitutes a major burden on affected children, their families and on the health system especially in communities with a high rate of consanguinity where incidence and prevalence of recessive inborn errors of immunity (IEI) are expected to be high.

**Method:**

Here, we report the clinical, immunological, and molecular findings in 36 children diagnosed with SCID from a single tertiary center in Oman for the last decade.

**Results:**

We observed a median annual incidence rate of 4.5 per 100,000 Omani live births, and 91.7% of affected children were born to consanguineous parents. Twenty-three children (63.9%) fulfilled the criteria for typical SCID. The median age at onset, diagnosis and diagnostic delay were 54, 135, and 68 days, respectively. The most common clinical manifestations were pneumonia, septicemia, and chronic diarrhea. Eleven children (30.6%) have received hematopoietic stem cell transplant (HSCT) with a survival rate of 73%. The most frequent genetic cause of SCID in this cohort (n = 36) was (RAG-1), encoding for recombination activating gene (n = 5, 13.9%). Similarly, Major histocompatibility complex type II deficiency accounted for (n = 5, 13.9%) of our cohort.

**Conclusion:**

Our report broadens the knowledge of clinical and molecular manifestations in children with SCID in the region and highlights the need to initiate newborn based screening program (NBS) program.

## Introduction

Severe Combined Immunodeficiency (SCID) is a genetically heterogeneous, which is almost always a lethal disorder of infancy. It is characterized by an arrest in T lymphocyte development with variable abnormalities in B and NK cells. Clinical presentation is dominated by severe opportunistic infections. More than twenty monogenic defects have been identified in children with SCID (1, 2). The incidence of SCID varies in different geographical locations. This reflects differences in prevalence of recessive disorders as well as differences in case ascertainment depending on whether newborn based SCID screening programs (NBS) have been implemented. Thus, the prevalence of SCID has been estimated at 1/100,000 in parts of the USA that have implement NBS (3), compared to 20/100,000 live births in the Middle East (4).

To date, a population based NBS diagnosis entails the best strategy for the early identification of such affected newborns prior to the onset of infections and other complications. In fact, the introduction of the NBS programs has shown that SCID is commoner than initially thought. SCID NBS has revealed an incidence of 1 in 131,485 in Taiwan (5), 1: 58,000 in USA (6), and 1 in 11,821 in China (7). However, in countries where consanguineous marriages are a common practice, the incidence have been reported to be higher such as in Saudi Arabia was found to be 1:2,906 (8), (20×) higher than the incidence reported from USA NBS programs.

SCID is often fatal if undiagnosed and untreated within the first 1–2 years of life. The main stay of treatment of SCID is hematopoietic stem cell transplantation (HSCT) with nearly normal survival at 5 years interval, particularly when HSCT is completed by the age of 3.5 months of life in children free from infections. Mortality increases sharply in older children with active infection at the time of HSCT, survival falls to 50–80% (9, 10).

Oman has a remarkably high rate of consanguineous marriage. Almost half (49%) of the total marriages being blood related (11). The estimated prevalence of primary immunodeficiency PID disorders in Oman is about 7 cases per 100,000 with a predominance of phagocytic disorders. While a previous report suggested a relatively low incidence of SCID in 10 out of 140 cohort described by AL Tamemi et al. (12), it is possible that this reflected incomplete case ascertainment. Here, we provide an up-to-date description of the clinical, immunological, and molecular findings and outcomes in children with SCID in a single tertiary center in Oman for the last decade.

## Methods

### Patients

We described a retrospective cohort of children confirmed to have SCID at the Royal Hospital—the main governmental tertiary hospital in Oman—from January 2010 to January 2020. The diagnostic criteria for SCID is based on the Primary Immune Deficiency Treatment Consortium (PIDTC) guidelines (13). Typical SCID: 1) absence or very low number of autologous T cells (CD3 T cells <300/ml) and 2) no or very low T-cell proliferation (<10% of lower limit of normal) as measured by response to PHA. Presence of T cells of maternal origin as an alternative diagnostic criterion when T-cell proliferation is not available. Atypical SCID: 1) Reduced number of CD3 T cells for age: up to 2 years <1,000/ml, >2 up to 4 years <800/ml, and for >4 years <600/ml. 2) Absence of maternal engraftment. 3) Reduced T-cell function to PHA < 30% of lower limit of normal. Additionally, molecular defects for MHC-II deficiency in class Ib, DiGeorge syndrome in class IIa, immunodeficiency with multiple intestinal atresia in class IIb and CD25 deficiency in class IVb listed in IUIS expert committee opinion on IEI-2019 has been included when presenting with typical or atypical SCID phenotype (14). Definitive diagnosis was made based on the molecular analysis when available.

We collected demographic and clinical details including gender, age of onset, age of diagnosis, diagnostic delay, consanguinity, family history of SCID, a presence of deceased sibling with SCID, geographical distribution, administration of BCG vaccine, infectious etiology, immunological workup, molecular workup and outcomes. Microbiological workup of invasive and non-invasive infections has been recorded including bacterial mycobacterial and fungal cultures, mycobacterium PCR and acid-fast bacillus analysis, plasma and fluid viral PCR, virus-specific inclusion bodies in histopathology, feces analysis for bacteriology parasitology and viral PCR and cerebrospinal fluid analysis for bacteriology and viral PCR when indicated. Infections were oral thrush, lower respiratory tract infections, bacteremia/sepsis, gastro-intestinal infections, viremia, cellulitis/skin abscess, ear infections, urinary tract infections, conjunctivitis, BCG-related infections and others.

### Immunological Evaluation

Workup included complete blood count, serum immunoglobulin levels (IgG, IgA, IgM, and IgE), immunization serological responses and lymphocyte subset analysis by flowcytometry (CD3, CD4, CD8, CD19, and CD56). Additionally, a detailed enumeration by flowcytometry of CD4, CD8 and CD19 compartments, quantitative analysis of recent thymic emigrants (RTE), T cell receptor Vβ repertoire analyses and lymphocyte proliferation response to phytohemagglutinin (PHA) have been requested for some children as the above tests were only available since 2017. Newborn screening using TRECs analysis is not available in Oman at present.

### Genetic Analysis

Whole exome sequencing by Centogene^®^ or targeted mutation analysis for those with positive family history of SCID has been requested for all children. The entire exome dataset has been evaluated for variants clinically relevant to the described phenotype. In addition, pathogenic or likely pathogenic variants has been identified. Further testing (structural genetic variants or CentoImmuno^®^ NGS panel) was perform as indicated. In some cases where a diagnosis was obtained, additional sequencing was performed at Centre for Personalised Immunology, Canberra, Australia.

## Results

### Patient Demographics

We identified children with SCID (n = 36) at the Royal Hospital between January 2010 and January 2020, yielding an annual incidence of 4.5 (range: 2.7–8.7) per 100,000 Omani live births. Twenty-one children were females (58.3%, M/F: 0.71) and 33 children (91.7%) were born to consanguineous parents. In twenty-four cases (66.7%) there a positive family history of SCID. In 12 cases (33.3%) there was a history of sibling death with SCID. The governorate with highest reported prevalence was AL-Sharqiya South (12 children, at 33.3%). This is followed by AL-Batina North and Muscat (7 children each, at 19.4%), AL-Dakhiliyah and AL-Dhahira (3 children each, at 8.3%), Dhofar (2 children, at 5.6%), and AL-Batina South and Musandam (1 child each, at 2.8%).

### Clinical Phenotype

**Figure 1:**
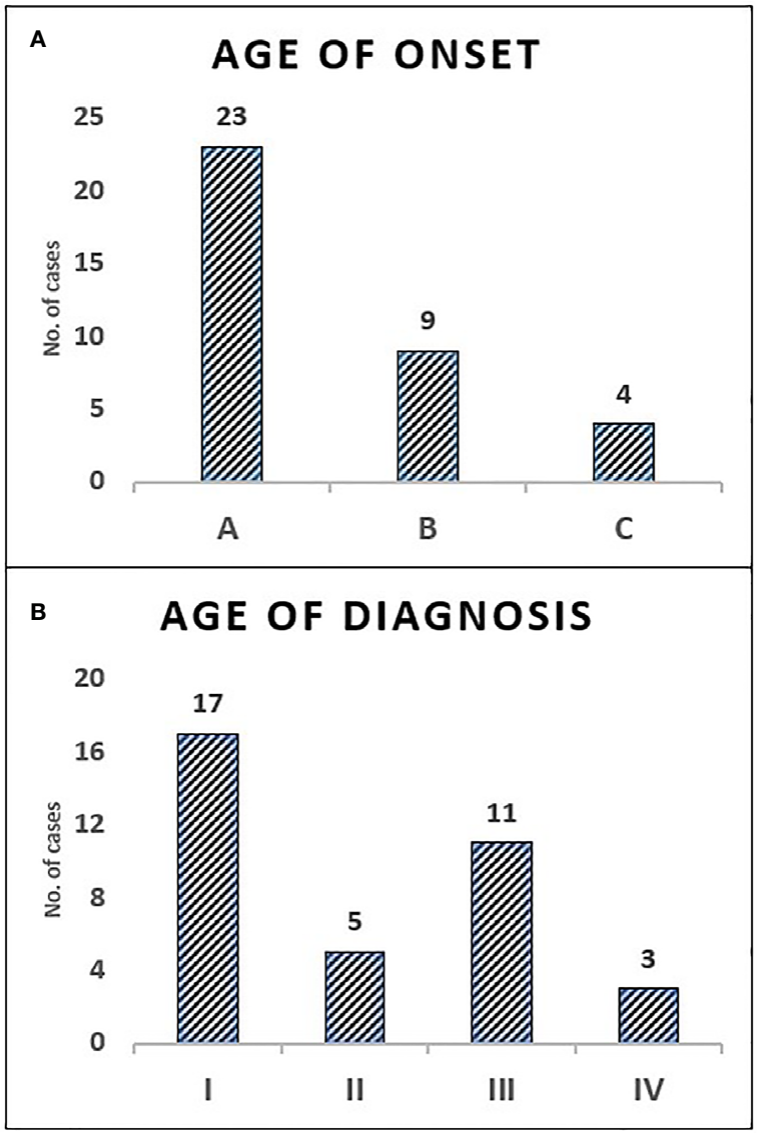
The distribution of children with SCID in categories of age—onset and diagnosis. Onset age category A: (0–3 months) 63.9%, B: (4–6 months) 25%, C: (7–12 months) 11.1% **(A)**. Diagnosis age category I: (0–3 months) 47.2%, II: (4–6 months) 13.9%, III: (7–12 months) 30.6%, IV: (>12 months) 8.3% **(B)**.

**Figure 2:**
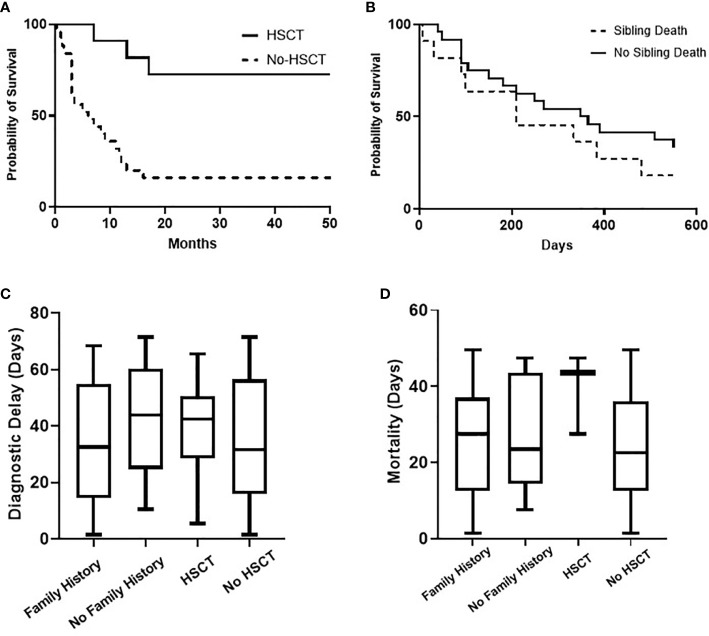
Overall probability of survival (Kaplan-Meier curve) of children with SCID for HSCT (P value = 0.0003) and for the presence of sibling death (P value = 0.2923), **(A, B)**. Comparative analysis (Kruskal-Wallis test) for diagnostic delay (P value = 0.5702) and mortality age (P value = 0.3427) in relation to family history and HSCT, **(C, D)**.

All children with SCID had classical features of the disease and 6 children (16.7%) fulfilled the criteria for Omenn syndrome. Twenty-three children (63.9%) have fulfilled the criteria of typical SCID (autologous CD3+ T cell count < 0.3 x 109/L) and developed manifestations of disease within the first three months of life. Remaining 9 children (25%) and 4 children (11.1%) have developed disease manifestation at 4–6 months and 7–12 months of age respectively. Nineteen children (53.8%) have been diagnosed with SCID after the third month of life (5 children at the age of 4–6 months, 11 children at the age of 7–12 months and 3 children over the age of 12 months) (**Figure 1**). The median age difference between the onset of symptoms and diagnosis (diagnostic delay) was 68 days. Medians for diagnostic delay in relation to family history and HSCT indicated no statistical significance (**Table 1**) (**Figure 2**). Most children (n = 34) received BCG vaccination at birth before the diagnosis. The most frequent infections were lower respiratory tract infection in 31 children (86.1%), septicemia in 21 children (58.3%), gastro-intestinal infections in 16 children (44.4%), symptomatic viremia in 12 children (33.3%), cellulitis/skin abscess in 7 children (19.4%), ear infections in 6 children (16.7%), BCG-related infections and conjunctivitis in 4 children each (11.1%), osteomyelitis and urinary infections in 3 children each (8.3%) and meningitis in 2 children (5.6%) (**Table 2**).

**Table 1:** Medians and P values (P < 0.05, CI 95%) for age of onset, age of diagnosis, diagnostic delay, and mortality in relation to HSCT and family history of SCID.

		Age of onset	Age of diagnosis	Diagnostic delay	Mortality age
HSCT	Median (days)	120	240	90	390
No HSCT		23	90	56	165
	P value	0.0514	0.1390	0.4816	0.0843
Family history	Median (days)	31.5	100	58	210
No family history		120	180	95	180
	P value	0.0724	0.1581	0.2337	0.7044

**Table 2:** Clinical features of 36 children with SCID phenotype.

Patient	Defect	CD3 (10^9/l)	Age-onset (months)	Age-diagnosis (months)	Outcome	Broncho-alveolar lavage	Septicaemia, CLABSI*	Gastro-intestinal infections	Viral pneumonitis, *viremia*	Other infections
P1	Interleukin-2 receptor gamma	0.00	0–3	0–3	Mortality		Pseudomonas aeruginosa			Skin abscess: Pseudomonas aeruginosa Enterococcus faecium
P2	Interleukin-2 receptor alpha	0.21	0–3	7–12	Mortality				*CMV*	Urine: Enterococcus fecalis
P3	SCID, unknown	0.00	0–3	4–6	Mortality	Pseudomonas aeruginosa Stenotrophomonas maltophilia Candida krusei Klebsiella pneumoniae	Staphylococcus epidermidis		Parainfluenza 3	Eye: Klebsiella pneumonia
P4	Recombination activating gene	0.02	0–3	4–6	HSCT, alive and well			Rota Virus		
P5	Major histocompatibility complex type II	3.86	7–12	7–12	HSCT, mortality	Pneumocystis jirovecii Pseudomonas aeruginosa CMV	Klebsiella pneumonia	Klebsiella pneumonia (colon) Escherichia coli (colon)	Rhinovirus Adenovirus *CMV* *Adenovirus*	Ear: Pseudomonas aeruginosa, Staphylococcus aureus, Acinetobacter lwoffi Skin abscess: Staphylococcus aureus
P6	Janus kinase 3	1.99	7–12	>12	Mortality		Escherichia coli Staphylococcus aureus *Staphylococcus aureus*	Rota Virus Salmonella species	Rhinovirus Parainfluenza 3	
P7	Recombination activating gene	0.02	4–6	7–12	Mortality			Salmonella species		
P8	Interleukin-2 receptor gamma	0.01	0–3	0–3	Mortality	Pneumocystis jirovecii Pseudomonas aeruginosa			RSV	
P9	Major histocompatibility complex type II	1.28	7–12	7–12	HSCT, alive and well	Pneumocystis jirovecii Pseudomonas aeruginosa Candida albicans Adenovirus	Pseudomonas aeruginosa	Enterovirus	Rhinovirus RSV Parainfluenza 3 Enterovirus Adenovirus	Urine: Candida albicans Bone: Pseudomonas aeruginosa
P10	Major histocompatibility complex type II	0.81	4–6	7–12	HSCT, mortality	Pneumocystis jirovecii Enterobacter cloacae MRSA Leclercia adecarboxylata		Clostridium difficile	Rhinovirus Enterovirus	
P11	CD3 epsilon	0.05	0–3	0–3	Mortality	Pneumocystis jirovecii CMV Candida spp Aspergillus niger			Adenovirus *Adenovirus* *CMV*	
P12	SCID, unknown	1.68	4–6	7–12	HSCT, alive and well	Pneumocystis jirovecii		Enterovirus	Rhinovirus	Skin abscess: Pseudomonas aeruginosa, Escherichia coli, Candida spp
P13	Interleukin-7 receptor	0.00	0–3	0–3	Mortality		Escherichia coli			Skin abscess: Escherichia coli
P14	Adenosine deaminase	0.00	0–3	0–3	Mortality	Pneumocystis jirovecii				
P15	Major histocompatibility complex type II	6.18	4–6	7–12	HSCT, alive and well	Pneumocystis jirovecii Haemophilus influenza Enterobacter cloacae CMV			Rhinovirus Bocavirus *CMV*	
P16	Interleukin-7 receptor	0.02	4–6	7–12	Mortality	Pseudomonas aeruginosa MRSA				
P17	Adenosine deaminase	0.00	0–3	0–3	Mortality	Mycobacterium simiae	Klebsiella pneumonia *Citrobacter werkmanii* *Klebsiella pneumonia*	Rota Virus	Rhinovirus RSV	Lung biopsy: Staphylococcus epidermidis
P18	Major histocompatibility complex type II	8.19	4–6	7–12	Mortality	Pneumocystis jirovecii Elizabethkingia meningoseptica		Adenovirus (colon)	Rhinovirus Parainfluenza 2 Enterovirus	
P19	Adenosine deaminase	0.00	0–3	0–3	Mortality	Stenotrophomonas maltophilia				
P20	Tetratricopeptide repeat domain-7A	0.51	0–3	7–12	Mortality	Pneumocystis jirovecii Stenotrophomonas maltophilia CMV Candida spp	Staphylococcus epidermidis	Salmonella species CMV (colon)	Rhinovirus *CMV* *Adenovirus*	
P21	SCID, unknown	0.01	0–3	0–3	Mortality		Staphylococcus haemolyticus Staphylococcus epidermidis			
P22	Janus kinase 3	0.00	0–3	4–6	Mortality	Pneumocystis jirovecii Mycobacterium bovis Candida albicans	*Klebsiella pneumonia*		Rhinovirus Bocavirus *HSV-1* *Sapovirus*	Eye: Haemophilus influenzae
P23	Cernunnos XLF	1.48	4–6	>12	Refused HSCT and lost follow up	Pseudomonas putida CMV EBV			*CMV* *EBV*	
P24	Adenosine deaminase	0.00	4–6	4–6	Mortality		Staphylococcus epidermidis Candida auris			PD site wound culture: Candida auris PD fluid culture: Candida parapsilosis
P25	Tetratricopeptide repeat domain-7A	0.57	0–3	0–3	Mortality		Staphylococcus epidermidis	CMV (colon)	*CMV*	
P26	Interleukin-7 receptor	0.02	4–6	7–12	HSCT, alive and well	Pneumocystis jirovecii CMV Adenovirus Mycobacterium bovis	Salmonella species Streptococcus salivarius	Rota Virus Mycobacterium bovis (colon)	Rhinovirus Parainfluenza 3 Bocavirus *CMV* *Adenovirus*	Eye: Salmonella species CNS PCR: Adenovirus Skin nodule culture and knee effusion: Mycobacterium bovis Skin abscess: Salmonella spp
P27	Interleukin-7 receptor	0.01	0–3	0–3	HSCT, mortality	Pseudomonas aeruginosa Candida albicans Cryptosporidium spp (PCR)	Enterococcus faecium Staphylococcus haemolyticus	Klebsiella pneumonia (colon) Enterococcus faecium (colon) Candida dubliniensis (colon) Candida tropicalis (colon)		Skin abscess: Staphylococcus aureus
P28	Recombination activating gene	0.00	0–3	0–3	Mortality	Adenovirus			Adenovirus Enterovirus *Adenovirus*	
P29	SCID, unknown	0.56	7–12	>12	HSCT, alive and well	CMV EBV	Staphylococcus epidermidis		RSV *CMV* *EBV*	
P30	Janus kinase 3	0.00	0–3	0–3	Mortality	Haemophilus influenza Stenotrophomonas maltophilia Candida albicans Mycobacterium bovis	Staphylococcus epidermidis Candida albicans Candida parapsilosis *Enterococcus faecium* *Staphylococcus epidermidis*	Rota Virus		Ear: Candida albicans Urine: Enterococcus genus
P31	CD3 epsilon	0.01	0–3	0–3	HSCT, alive and well		Staphylococcus epidermidis Staphylococcus hominis		Rhinovirus Parainfluenza 3 *EBV*	BCG adenitis: Mycobacterium bovis Eye: Haemophilus influenzae
P32	SCID, unknown	0.50	0–3	4–6	Mortality	Pneumocystis jirovecii	Staphylococcus epidermidis Pseudomonas diminuta		RSV Influenza A	
P33	Complete 22q11.2 DS	0.16	0–3	0–3	Alive with complications					
P34	Complete 22q11.2 DS	0.04	0–3	0–3	Undergoing thymic transplantation, alive with complications	CMV			Rhinovirus CMV	
P35	Recombination activating gene	0.00	0–3	0–3	HSCT, alive and well					
P36	Recombination activating gene	8.8	0–3	0–3	Mortality		Staphylococcus haemolyticus Staphylococcus epidermidis			Ear: Pseudomonas aeruginosa

CD3, cluster of differentiation 3; SCID, severe combined immunodeficiency; CMV, cytomegalovirus; EBV, Epstein Barr virus; RSV, respiratory syncytial virus; HSCT, hematopoietic stem cell transplant; CLABSI in Italic*, central line-associated bloodstream infection.

### Immunological Evaluation

All children underwent basic immunological assessment at the time of presentation. Twenty-seven children (75%) had lymphopenia (<2,000 cell/ul) at the first encounter. Seven children have had elevated serum IgE, six children of those with Omenn syndrome. Median lymphocyte subset count for CD3: 0.02 (10^9/l), CD4: 0.015, CD8: 0.005, CD19: 0.57, and CD56: 0.09. Median serum immunoglobulin levels for IgG: 1.30 (g/l), IgA: 0.00 and IgM: 0.00. All 15 children (41.7%) who were tested for T cell proliferation response to mitogen and RTE enumeration had absent proliferative responses to PHA and significantly reduced RTE population.

### Genetic Analysis

A molecular diagnosis was made in 31 children (86.1%). Pathogenic RAG-1 variants were found in 5 children (13.9%). Five children (13.9%) were identified with a defect in MHC class II expression—bare lymphocyte syndrome (BLS), and this was accounted for by variants in CIITA (n = 3), RFXANK (n = 1), and RFX5 (n = 1). Adenosine deaminase (ADA) deficiency in 4 children (11.1%), interleukin-7 receptor (IL-7R) deficiency in 4 children (11.1%), janus kinase-3 (JAK-3) deficiency in 3 children (8.3%), interleukin-2 receptor gamma (IL-2RG) deficiency in 2 children (5.6%), CD-3 epsilon (CD3E) deficiency in 2 children (5.6%), tetratricopeptide repeat domain-7A (TTC7A) deficiency in 2 children (5.6%), complete 22q11.2 DS in 2 children (5.6%), non-homologous end-joining factor-1/Cernunnos XLF deficiency in 1 child (2.8%) and interleukin-2 receptor alpha (IL-2RA) deficiency in 1 child (2.8%) (**Table 3**).

**Table 3:** Genotype summary of 36 children with SCID.

Defect	Gene	Patient	Mutation	Protein effect	Exon	Type	Zygosity
Recondition activating gene	RAG1 RAG1	4,28,36 7,35	c.1187G>A c.2924G>C	p.(Arg396His) p.(Arg975Pro)	2 NA	Missense Missense	Homozygous Homozygous
Major histocompatibility complex type II	CIITA CIITA RFXANK RFX5	9,10, 18 5 15	c.3215T>C Chr16:10907596-10907803del c.634C>T c.446G>A	p.(Met1072Thr) GRCH38 p.(Arg212Ter) p.(Arg149Gln)	17 NA 9 NA	Missense NA Nonsense NA	Heterozygous Homozygous Homozygous Homozygous
Interleukin-7 receptor	IL7R	6,13,16, 27	c.616C>T	p.(Arg206)	5	Nonsense	Homozygous
Adenosine deaminase	ADA ADA ADA	17 14,19 24	c.910del c.815G>A c.646G>A	p.(Leu304Trpfs7) p.(Trp272) p.(Gly216Arg)	10 NA 7	Frameshift Stop gain Missense	Homozygous Homozygous Homozygous
Janus kinase 3	JAK3 JAK3 JAK3	30 30 6,22	c.2490+1G>A c.1645C>T c.1613G>A	NR p.(Arg549) p.(Gly538Asp)	18 NA 12	Splicing Nonsense Missense	Compound Heterozygous Compound Heterozygous Homozygous
CD3 epsilon	CD3E	11,31	c.351A>C	p.Arg117Ser	6	NA	Homozygous
Interleukin-2 receptor gamma	IL2RG	1, 8	c.854G>A	p.Arg285Gln	6	NA	Hemizygous
Tetratricopeptide repeat domain-7A	TTC7A	20,25	c.122del	p.(Met41Serfs38)	1	Frameshift	Homozygous
Cernunnos XLF	NHEJ1	23	c.530-1G>A	Intron 4	5	Substitution	Homozygous
Interleukin-2 receptor alpha	IL2RA	2	c.418 T>C	aa.Y140H	4	NA	Homozygous

NA, not available.

### Management and Outcome

All children received antimicrobial prophylaxis and regular intravenous immunoglobulin therapy at the time of diagnosis. Eleven children (30.6%) with a genetic diagnosis of: RAG1 (n = 2), CIITA (n = 2), IL7R (n = 2), no genetic diagnosis (n = 2), RFXANK (n = 1), RFX5 (n = 1), and CD3E (n = 1) received HSCT with a median transplant age of 12 months. Median transplant age for children with family history of SCID (n = 6) was 9 months. Whereas it was 16 months for those with no family history of SCID (n = 5). For those who received HSCT, 8 children are currently alive with no complications while 3 children died during or just after transplant. Four of eight successful HSCT were from matched-sibling donors, while the others were accounted for by family-matched (n = 3) and haploidentical donor (n = 1). All the 3 children who died after HSCT received from haploidentical stem cells, their age of HSCT was >3.5 months, and they had active infections at the time of transplantation. Probability of survival for children with SCID in relation to HSCT and presence of sibling death is shown (**Figure 2**). A child with complete DiGeorge syndrome (P34) was recently enrolled in thymic transplantation and a second child (P33) is planned for the same. Parents of child (P23) elected not to pursue HSCT and lost follow up, hence the outcome is unknown. Twenty-two children (61.1%) with SCID died before HSCT. Thus, overall mortality was in 25 children (69.4%) with a median mortality age of 210 days. Medians for mortality age in relation to family history of SCID and HSCT indicated no statistical significance (**Table 1**) (**Figure 2**).

## Discussion

Sultanate of Oman is a country on the southeastern coast of the Arabian Peninsula in Western Asia. As of September 2020, Oman’s population is over 5 million with a total fertility rate of 2.9 (15). As in neighboring countries, there is a high rate of reported consanguineous marriages among Omani population reaching to almost 49%. This has contributed to high frequency of congenital genetic diseases in the country (16) and invariably results in increasing burden of autosomal recessive conditions causing IEI. In 2016, AL Tamimi S et al. estimated the PID population prevalence at 7.0 for every 100,000 Omani live births (12) but identified only 10/140 children with SCID suggesting a possibility of suboptimal ascertainment.

The accurate incidence of SCID amongst the Omani population is unknown. There is no national NBS program and therefore only a minority of children are detected before the onset of disease manifestations. We provide the first retrospective comprehensive national report from Oman that describes the clinical, laboratory, and molecular findings, and outcome for 36 children with SCID in a single tertiary center. We report a median annual incidence rate of 4.5 children with SCID per 100,000 Omani live births. As compared to incidences extrapolated from NBS programs, our incidence rate is lower than in China (1 in 11,821) (7), comparable with Israel (1 in 22,500) (17), and higher than US, Sweden and meta-analyses of 13 studies with incidence rate of ~1 in 58 thousand live birth (6, 18, 19).

This high incidence reflects the high rates of consanguinity in Oman. Ninety-one percent of our cohort were a product of consanguineous marriage. This is even greater than reported in large SCID cohorts from Iran (87.3%), KSA (60%) and India (36%) (8, 20, 21). Remarkably, in our cohort, 66% of affected children had a positive family history of SCID for which half of them (33%) reported a sibling death, much higher than what have been reported in Italy (5.6%) and Iran (3%) (20, 22).

Lymphopenia was observed in 75% of our children and majority of them have fulfilled the diagnostic criteria for typical SCID. However, some of our children presented with an atypical SCID phenotype displaying higher autologous CD3+ T cell counts and/or oligoclonal TCR pattern with a later-onset of infections. Atypical presentations contributed to delayed diagnosis in some cases. Severe pneumonia, bacterial and candida septic shock and diarrhea were the commonest presenting infections whereas a third of our cohort has presented with serious viral infections. As expected, we observed in our cohort a spectrum of polymicrobial infections with bacterial, viral, fungal, and protozoal organisms. It is well known that BCG vaccine has an exceedingly high rate of complications in children with SCID (23). While BCG is administered routinely at birth, only 11% of our cohort developed BCG-related complications. It is possible that most children died before the development of obvious symptoms and signs of BCG-related disease. Saudi Arabia have recently suggested a delay in administering the BGC vaccine by 6-month of age, to avoid the infective complications of BCG pending the diagnosis of PID. However, Oman have joined the international efforts with the world health organization (WHO) to develop strategies for TB elimination (24, 25). While the incidence of BCG-related complications is lower in our cohort, these cases still indicate that universal neonatal BCG vaccination carries a risk on the absence of concurrent NBS program for SCID.

The spectrum of genetic defects in our cohort revealed a wide genetic heterogeneity and a predominance of autosomal recessive causes. The most common gene implicated in our cohort was RAG-1 (phenotype of T-B-NK+). T-B-NK+ SCID phenotype was also observed to be prominent in communities with high consanguinity rates (8, 20, 26, 27). Interestingly, we identified five cases of BLS resulting from defects in CIITA, RFXANK and RFX5. Few children had no pathogenic nor likely-pathogenic variant explaining the SCID phenotype. However, HSCT resulted in a favorable outcome for (P29) and should be a preference in such situations.

SCID is a pediatric emergency and affected children can only be rescued by HSCT and for some types by using gene therapy (GT) or enzyme replacement therapy (ERT) as indicated. ERT is not available in Oman at present and therefore not an option for children with ADA. The overall outcome in this cohort was poor with mortality occurring in 69% of the children at a median age of 7 months. Less than a third of our cohort underwent HSCT and the mean transplant age at transplant was 12 months. Further delay in transplant age for children with no family history of SCID (n = 5) to 16 months highlights the importance of early detection. Thus, most children succumbed before HSCT because of diagnostic delay, severe infections and related complications and complex phenotype. Of those undergoing HSCT, a successful outcome was observed in 73% of cases and although the numbers were small, we observed better outcome in children with no or minimum infection at the time of transplant and in whom the donors were HLA matched either from a sibling or a family relative. In contrast, death occurred in those who had active infection and underwent haploidentical transplant.

These outcome data emphasize the problems of a delay in diagnosis. Most children were referred to the clinical immunology unit for further workup and treatment only upon the appearance of clinical symptoms and signs suggestive of SCID. This had led to a delay in diagnosis, presentation with a complex phenotype, polymicrobial infections, hindrance of effective initiation of therapy and worse outcomes. The median interval of diagnostic delay was 2.3 months which is close to the reported data from Iran (2 months), India (3months), China (2.6 months), and Netherland (2months) (20, 21, 28, 29). Despite a third of our cohort had sibling death with SCID, the current detection strategy looks inadequate to ensure a better outcome as depicted in (**Table 1**) and (**Figure 2**). The diagnostic delay and hence poor outcome observed in this cohort reflect the need to improve awareness about the seriousness of SCID among the health care professionals and the community.

A national NBS program will help to identify the true incidence of SCID in Oman and reveal the autosomal recessive causes of SCID in the community (30). We expect that a substantial improvement in outcome for children with SCID would be observed with implementation of such program.

## Conclusion

This is the first comprehensive report that provides insight to the clinical, laboratory (including molecular) and outcome of children with SCID in Oman. High mortality is due to diagnostic delay, complex phenotype, polymicrobial infections at the diagnosis, late access to immune reconstitution therapy, and the unavailability of NBS program. The results of this study will help our health care authorities to recognize the seriousness of the health problem and to provide all required actions including redirecting the resources for the benefit of diagnosis and management of children with SCID. Measures that might improve outcomes for children with SCID in Oman include establishing awareness programs, the development of a local genetic panels for faster diagnosis of SCID, early access to HSCT and the initiation of NBS. We recommend having a future pilot study using NBS program in areas prevalent with SCID such as AL-Sharqiya South or AL-Batina North to assess the need and the cost-effectiveness of a forthcoming NBS program for SCID in Oman. Deferral of BCG vaccine until 6 months of age should be considered until NBS is implemented.

## Data Availability Statement

The original contributions presented in the study are included in the article/supplementary materials. Further inquiries can be directed to the corresponding author.

## Ethics Statement

The studies involving human participants were reviewed and approved by Scientific Research Committee. Written informed consent from the participants’ legal guardian/next of kin was not required to participate in this study in accordance with the national legislation and the institutional requirements.

## Author Contributions

NA: Conception and design, data collection, data analysis and interpretation, drafting the manuscript, critical revision. KA: Conception and design, data collection. JA: Immunophenotyping, drafting the manuscript. MA: Immunophenotyping, drafting the manuscript. MC: Genetic diagnosis, drafting the manuscript. TA: Conception and design, data collection, data analysis and interpretation, drafting the manuscript, critical revision. All authors contributed to the article and approved the submitted version.

## Conflict of Interest

The authors declare that the research was conducted in the absence of any commercial or financial relationships that could be construed as a potential conflict of interest.
